# Functional Connectivity and Complexity in the Phenomenological Model of Mild Cognitive-Impaired Alzheimer's Disease

**DOI:** 10.3389/fncom.2022.877912

**Published:** 2022-06-06

**Authors:** Surya Das, Subha D. Puthankattil

**Affiliations:** Department of Electrical Engineering, National Institute of Technology, Calicut, India

**Keywords:** functional connectivity, complexity, Kuramoto model, MCI-AD, EEG

## Abstract

**Background:**

Functional connectivity and complexity analysis has been discretely studied to understand intricate brain dynamics. The current study investigates the interplay between functional connectivity and complexity using the Kuramoto mean-field model.

**Method:**

Functional connectivity matrices are estimated using the weighted phase lag index and complexity measures through popularly used complexity estimators such as Lempel-Ziv complexity (LZC), Higuchi's fractal dimension (HFD), and fluctuation-based dispersion entropy (FDispEn). Complexity measures are estimated on real and simulated electroencephalogram (EEG) signals of patients with mild cognitive-impaired Alzheimer's disease (MCI-AD) and controls. Complexity measures are further applied to simulated signals generated from lesion-induced connectivity matrix and studied its impact. It is a novel attempt to study the relation between functional connectivity and complexity using a neurocomputational model.

**Results:**

Real EEG signals from patients with MCI-AD exhibited reduced functional connectivity and complexity in anterior and central regions. A simulation study has also displayed significantly reduced regional complexity in the patient group with respect to control. A similar reduction in complexity was further evident in simulation studies with lesion-induced control groups compared with non-lesion-induced control groups.

**Conclusion:**

Taken together, simulation studies demonstrate a positive influence of reduced connectivity in the model imparting a reduced complexity in the EEG signal. The study revealed the presence of a direct relation between functional connectivity and complexity with reduced connectivity, yielding a decreased EEG complexity.

## Introduction

Alzheimer's disease (AD) is one of the most common neurodegenerative diseases affecting the elderly population (Jeong, 2004). Electroencephalogram (EEG) studies, especially non-linear dynamics, are gaining popularity as a potential tool for the early detection of AD. An early diagnosis of the disease could aid in early interventions against the disease, subsequently increasing the quality of life. The three hallmark features from the non-linear/linear analysis of EEG signals of patients with AD are the slowing of EEG signals and reduction of functional connectivity and complexity (Dauwels and Cichocki, 2011). Recent studies with EEG signals have revealed the presence of reduced functional connectivity (Das and Puthankattil, 2020) and reduced complexity in the early stages of AD [mild cognitive-impaired AD (MCI-AD)] (Nimmy John et al., 2018).

Functional connectivity represents the correlation of neural activity among different brain regions through statistical interdependence measures. It provides the indices for functional integration between segregated cortical regions and has been correlated with aging (Varangis et al., 2019), learning (Veroude et al., 2010), and neurological disorders (Orekhova et al., 2014; Engels et al., 2015; Sargolzaei et al., 2015). The concept of complexity could be interpreted in different ways. Commonly used EEG complexity measures explain the complexity as a measure of the degree of randomness or degree of freedom associated with the system. However, complex behavior in a non-linear system could be exhibited with fewer degrees of freedom (Wackerbauer et al., 1994). Generally, a high and low entropy (i.e., random and regular order) system would have lower complexity, and an intermediate system would have higher complexity (Wackerbauer et al., 1994; Tononi et al., 1998).

A reduction in connectivity and complexity in MCI-AD/AD could be attributed to atypical non-linear neurodynamics in the brain (Jeong, 2004). Atypical non-linear dynamics in MCI-AD/AD brain could arise from pathophysiological changes due to the presence of tangles, alteration in synaptic couplings, and neuronal death (Nestor et al., 2004; Hornero et al., 2009). The AD brain has shown the presence of modest degrees of lesions with medial temporal lobe atrophy as a significant indicator in multiple studies (Visser et al., 2002; Clerx et al., 2013; Dhikav et al., 2014). Structural and functional connectivity studies in AD have revealed a reduction in the connectivity between different regions of the brain, converging into a network disconnection hypothesis (Delbeuck et al., 2003; Brier et al., 2014; Kundu et al., 2019). The disconnection hypothesis explains a neurodegenerative model with edges in the network model displaying a reducing trend of connectivity strength (Brier et al., 2014). Several research studies have related the reduction in the complexity measures to the decreased cortical connectivity, resulting in the diminished flexibility of the neural system to reach different information processing states (Babiloni et al., 2004; Al-nuaimi et al., 2018; Nobukawa et al., 2019). However, studies exploring the relation between complexity and functional connectivity are limited. This article attempts to explore the relation between functional connectivity and complexity using the Kuramoto mean-field model in the MCI-AD condition. The study also utilizes a lesion model to examine its impact on the network dynamics.

The Kuramoto model is a popularly used neurocomputational model based on weakly coupled limit-cycle oscillators. Nodes of the networks are defined by differential equations and edges by the cortical connectivity. The dynamics of the model could simulate data that have physiological properties similar to macroscopic features found in neurophysiological signals like EEG (Breakspear et al., 2010). One of the major advantages of the neurocomputational model based on Kuramoto would be the direct utilization of the connectivity matrix. One of the common applications of the Kuramoto model is in analyzing the structural–functional correlation of the brain to understand cortical dynamics (Finger et al., 2016; Lee and Frangou, 2017). The Kuramoto model has also been applied to studies on anesthesia (Schartner et al., 2015), consciousness (Ibáñez-molina et al., 2018; Lee et al., 2019), mind wandering (Ibáñez-molina et al., 2016), lesion (František et al., 2015; Jos et al., 2018), and complexity (Escudero et al., 2015). The studies generally used diffusion tensor tractography data for the connectivity pattern in the Kuramoto model (Escudero et al., 2015; Jos et al., 2018; Lee et al., 2019). The current study proposes to utilize functional connectivity data instead of structural connectivity data in the Kuramoto model to study the relationship between functional connectivity and complexity. Previous studies have utilized the Kuramoto model to study the relation between structural connectivity and complexity (Jos et al., 2018). We have used the Kuramoto model to validate the reduction of functional integration caused by the pathological process in MCI-AD, which would result in the reduction of the complexity score. The computational study also uses a lesion model in which edges originating from one specific region of the cortex are set to the lowest value. Lesions preferentially in the central part of a network could be a possible network lesion model in developing a neurocomputational model of AD (Aerts et al., 2016). The lesion model is exploited to study the impact of reduced connectivity with the complexity parameter.

**Figure 1:**
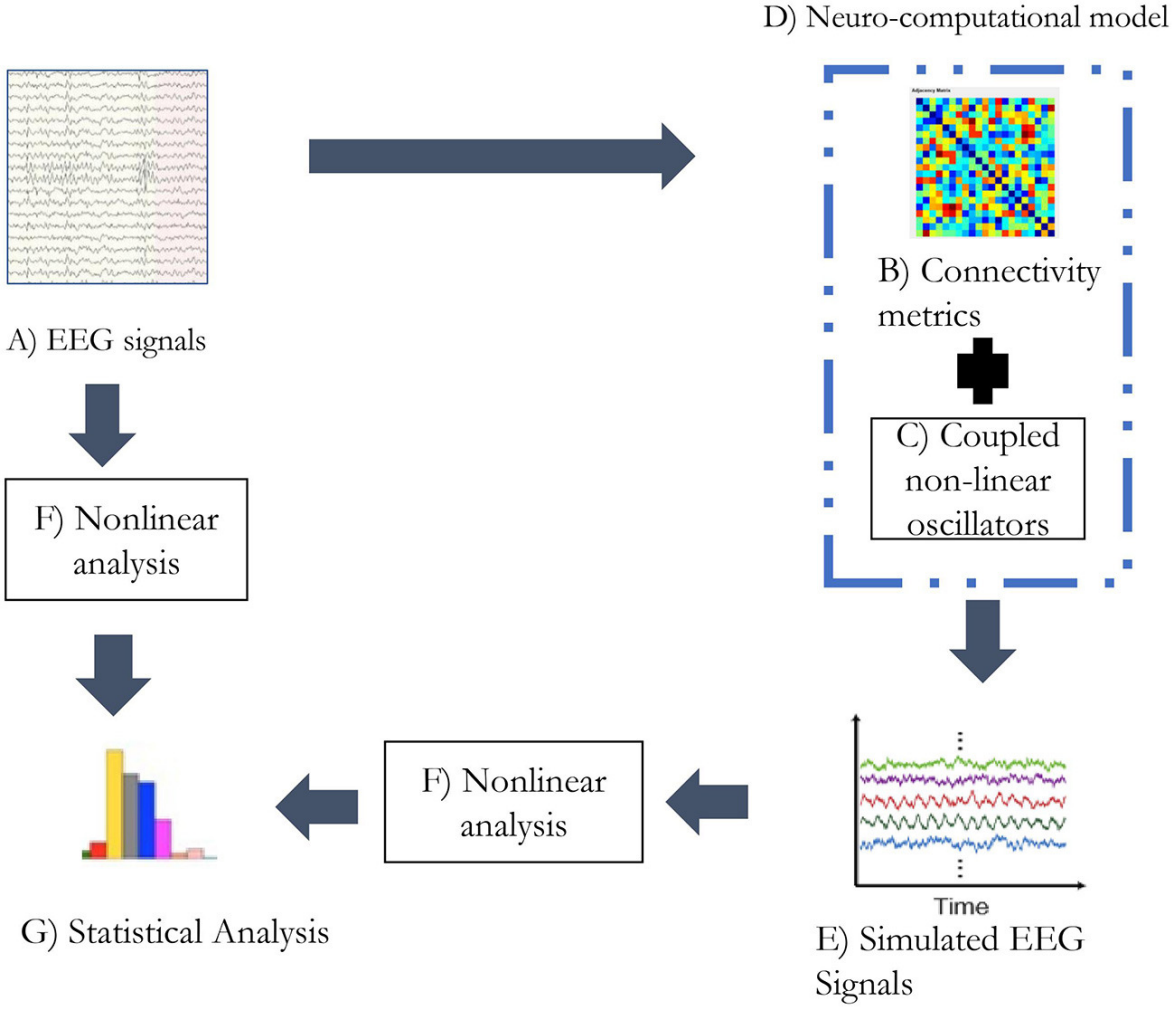
The analytic plan followed in the study. **(A)** The alpha band of the EEG signals was extracted through wavelet transform. **(B)** The connectivity matrix was generated from the alpha band of EEG signal utilizing weighted PLI. **(C)** Kuramoto's mean-field model was simulated with 21 oscillators. **(D)** Functional connectivity matrix and coupled non-linear oscillators form the neurocomputational model used in the study. **(E)** Simulated EEG signals being generated from the neurocomputational model. **(F)** Non-linear analysis was performed with metrics: LZC, HFD, and FDispEn. **(G)** Statistical analysis was performed on the estimates of non-linear analysis on real and simulated EEG signals separately.

Electroencephalogram studies reveal a gradual reduction in connectivity and complexity with the progression of the disease. This study analyzes the occurrence of the possible relation between connectivity and complexity in the context of the EEG signal simulation. It could be elucidated that reduction of connectivity could result in relatively isolated neural activities in the cortex that could influence the complexity of the system. This study explores the relation between connectivity and complexity through simulated EEG signals generated from the functional connectivity matrix of patients and controls. Complexity metrics were applied to real and simulated EEG signals to study the variation. The study also simulated EEG signals from the connectivity matrix with an induced lesion to study its characteristics. The steps followed in the study are represented in the form of a flowchart in Figure 1. The functional connectivity matrix from EEG signals was estimated using the weighted phase lag index (WPLI) (Vinck et al., 2011). WPLI is an efficient connectivity measure that indices the phase relation between brain regions with minimal interference through volume conduction. The complexity analysis was performed using Lempel-Ziv complexity (LZC), Higuchi's fractal dimension (HFD), and fluctuation-based dispersion entropy (FDispEn), where LZC and HFD are commonly used EEG complexity measures. FDispEn is a recent approach to measure dynamic variability in the fluctuation of EEG signals.

## Methodology

### Data Collection

Electroencephalogram data for the analysis were collected from 15 healthy controls and 13 subjects with MCI-AD. The sample population consists of participants from both genders in the age group of 57–75 years. Clinical dementia rating (CDR), Mini-Mental Scale Examination (MMSE), and Addenbrooke's cognitive examination (ACE) were used to rate dementia in MCI-AD. Patients with MCI-AD with the CDR score ≤1 were selected for the study. The mean and standard deviation of the various parameters of the sample population along with a significant difference between the groups considered in the study are provided in Table 1. Data acquisition was carried out at Sree Chithra Tirunal Institute of Medical Sciences and Technology, Trivandrum, Kerala, India. Ethical committee sanction was accorded for the study. Written and informed consent from patients and controls were obtained for the study.

**Table 1:** Mean and standard deviation of parameters in control and patient with mild cognitive-impaired Alzheimer's disease (MCI-AD).

	**Sample size (*n*)**	**Age (years)**	**Sex**	**MMSE**	**ACE**
Control	15	65.18 ± 3.15	7 males, 8 females	29.31 ± 1.03	92.47 ± 4.76
Patient	13	67.78 ± 6.10	7 males, 6 females	23.92 ± 4.15	63.85 ± 8.45
Significant difference	–	*p* = 0.09	–	*p* = 0.0001	*p* = 0.0002

Electroencephalogram data were acquired through a 32-channel digital acquisition system (NicVue, Nicolet-Viking, USA). EEG data from 21 electrode locations (Fp1, Fp2, F3, F4, C3, C4, P3, P4, O1, O2, T1, T2, F7, F8, T3, T4, T5, T6, Fz, Cz, and Pz) were preprocessed using simultaneous low-pass filtering and total variation denoising (LPF/TVD) algorithm (Selesnick et al., 2014). The signals were recorded with a sampling frequency of 400 Hz. EEG data were recorded for a duration of 5 min in the eyes open resting state. For the targeted lesion study, EEG data channels were clustered into three groups, namely, anterior (i.e., Fp1, Fp2, F7, F3, Fz, F4, and F8), central (i.e., T3, C3, Cz, C4, and T4), and posterior (i.e., T5, P3, Pz, P4, T6, O1, and O2). The alpha band of the EEG signals was extracted using wavelet transform. Wavelet transform is a multi-resolution decomposition method. The technique requires the selection of an appropriate wavelet function for the signal to be decomposed into different frequency scales. Wavelet “db10” was used in this study as it has given a good correlation coefficient with most of the signals acquired from the sample population. The functional connectivity analysis is performed through WPLI as a connectivity measure. The connectivity matrix thus generated was then fed into the Kuramoto model for generating simulated EEG signals. Complexity measures of LZC, HFD, and FDispEn were applied in real EEG signals and simulated EEG signals to study the variation.

### Functional Connectivity

Weighted phase lag index (Vinck et al., 2011) is the weighted version of PLI. PLI is a measure to quantify phase synchronization and indexes, the asymmetry of the distribution of relative phase around zero. It is motivated by the fact that non-zero phase difference occurs only through the result of true interaction. Thus, the measure is invulnerable to volume conduction and depends only on the phase difference. To increase the sensitivity to small signals and to mitigate the effect of noise, Vinck et al. have proposed some adjustments in PLI, yielding WPLI. In WPLI connectivity measure, phase leads or lags are weighted by the magnitude of the imaginary part of the complex cross-spectrum.


(1) $$WPLI_{xy}\;=\;\frac{\frac{1}{n}\sum {}_{t=1}^{n}\left|imag\left(S_{xyt}\right)\right|sgn(imag(S_{xyt}))}{\frac{1}{n}\sum {}_{t=1}^{n}\left|imag(S_{xyt})\right|}$$

In this equation, *S*_*xy*_ indicates the cross-spectral density between *x* and *y* time series data at time point *t* in the complex plane *xy*. *Sgn* is the sign function (−1, +1, or 0).

### Kuramoto Model

The Kuramoto model is used to mimic the dynamics of synchronization of activity between brain regions of MCI-AD and control. The model consists of a set of coupled differential equations. The model defines the dynamics of *N* identical oscillators.


(2) $$\begin{array}{r} \frac{\text{d}\theta_{\text{i}}}{\text{dt}}\;=\;\omega_{\text{i}}+\text{k}\overset{\text{N}}{\underset{\text{j }=\;1}{\sum }}{\text{a}}_{\text{ij}}\text{sin}\left(\theta_{\text{j}}-\theta_{\text{i}}\right) \end{array}$$

where *N* is the number of oscillators (nodes) in the model. Each node is equated to different electrode locations in the brain. θ_*i*_is the phase of *i*^*th*^ oscillator on its limit cycle. In this study$,\;\left(\frac{d\theta_{i}}{dt}\right)$ represents the rate of change of the phase of *i*^*th*^ oscillator. The variables ω and *k* denote the natural frequency and coupling strength of the oscillator network, respectively. The behavior of the system is strongly determined by the parameter *k*. When the system has *k* >*k*_*critical*_value, the system reaches a state of global synchrony. Similarly, when *k*<*k*_*critical*_ value, the system exhibits a low value of global synchrony. Thus, *k*_*critical*_ defines the bifurcation in the system dynamics. When *k* is poised near the *k*_*critical*_value, the system displays complex behavior. The variable *a*_*ij*_ denotes the connectivity matrix.

The degree of synchrony in the coupled oscillators can be measured through an order parameter *r(t)*.


(3) $$\begin{array}{r} \text{r}\left(\text{t}\right){\text{e}}^{-i\psi \left(\text{t}\right)}\;=\;\frac{1}{\text{N}}\overset{\text{N}}{\underset{\text{j }=\;1}{\sum }}{\text{e}}^{{-i\theta }_{j}\left(\text{t}\right)} \end{array}$$

The order parameter takes the value from 0 to 1 and measures the phase coherence of *N* oscillators. The order parameter of 1 represents perfectly synchronized oscillators and 0 represents perfectly unsynchronized oscillators. The symbol ψ represents the average phase of collective oscillators.

### EEG Simulation

**Figure 2:**
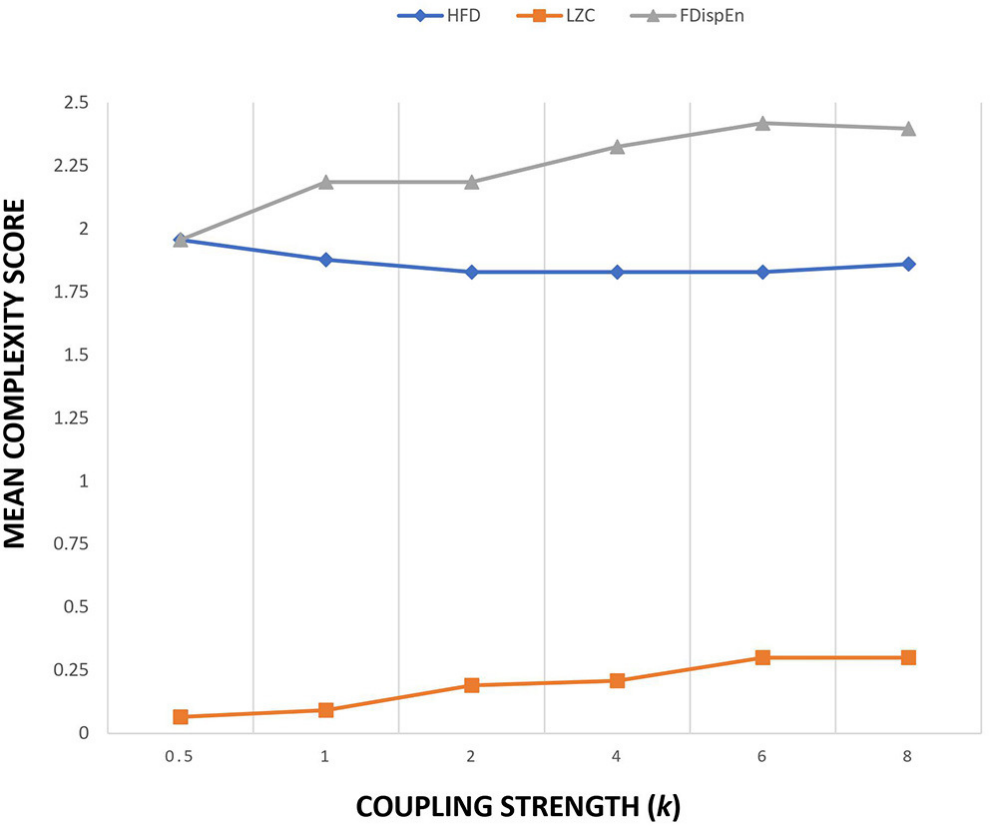
Sensitivity plot of three complexity measures as a function of coupling strength k of Kuramoto order parameter.

The Kuramoto model implemented in this study uses 21 oscillators to simulate 21-channel electrode locations. The connectivity matrix obtained from WPLI measure on 21 channel EEG signals is used as the connection strength *a*_*ij*_. WPLI measure was extracted from the alpha band of patients and controls during the eyes open protocol. WPLI operates on phase space and estimates maximally weighting ±90° phase difference between different EEG channels. It essentially detects phase lag interactions from a complex coupled system like the brain. Since the connection strength matrix *a*_*ij*_ is the connectivity strength estimated from the functional connectivity data instead of the structural connectivity data, the value of coupling strength *k* is kept constant at 1. Figure 2 provides the sensitivity plot between the mean complexity measure and coupling strength. A variation in the coupling value of *k* in the Kuramoto model alters the global connectivity strength. Complexity measures used are LZC, HFD, and FDispEn. From the sensitivity plot, the complexity estimates were nominally altered by the change in the coupling parameter. To ensure uniformity among the simulation studies for conducting a comparative analysis, the coupling strength was maintained at a constant value of 1. Since the Kuramoto model utilized in the study uses functional connectivity data, the data matrix is also inclusive of the bias from time delay, the weighted contribution from different sources, and the weighted reduction while passing through different layers of the brain.

The simulation model uses a frequency range (ω) that matches the frequency of the alpha band. The alpha band was specifically chosen in this study as this band was significantly affected in multiple functional connectivity studies conducted in AD/MCI-AD (Miraglia et al., 2016; Afshari and Jalili, 2017; Das and Puthankattil, 2020; Duan et al., 2020). The natural frequency of the coupled oscillators ($f_{i}\;=\;\frac{\omega_{i}}{2\pi }$) is randomly assigned with the distribution of mean and standard deviation of 10 Hz and 2 Hz, respectively.


(4) $$\begin{array}{r} {\text{x}}_{\text{i}}\left(\text{t}\right)\;=\;\overset{\text{N }=\;21}{\underset{\text{i }=\;1}{\sum }}\text{sin}\theta_{\text{i}}\left(\text{t}\right) \end{array}$$

In this study, *x*_*i*_(*t*) is the simulated EEG signal. The model was simulated to produce 15,000 sample points with Euler's integration scheme of *h* to be 0.1. Initial 1,000 sample points were discarded as the initial condition. The simulation is repeated 15 times with connectivity matrices of patients with MCI-AD, healthy controls, and lesion-induced controls. The connectivity matrices were averaged across patients with MCI-AD, healthy controls, and lesion-induced controls.

### Lesion Model

The popular hypothesis explaining AD pathology is the network disconnection model (Brier et al., 2014). For progressive neurodegenerative diseases such as Alzheimer's, the hub regions are preferentially vulnerable to lesions (Aerts et al., 2016). Hubs in the topologically central regions were likely to be more vulnerable to a pathological process like AD. In this study, the lesion in the topologically central region of the network is simulated by the reduction of connectivity strength. Thus, the lesion simulates a transformed network with limited edge strength, specifically in the topological central region. Edges originating in the central region of the brain (i.e., T3, C3, Cz, C4, and T4) were replaced with constant edge strength of 0.1 to simulate the effect of a lesion. The introduction of the lesion could significantly alter the functional dynamics, possibly influencing the complexity score of the system. The constant edge strength of 0.1 was specifically chosen as it is the lowest non-zero connectivity strength in the averaged connectivity matrix over the patient population. The connectivity strength in the averaged patient matrix varied between 0.1 and 0.6. The selection of 0.1 edge strength could accentuate the difference between the matrices. This resulted in the reduction of the mean connectivity strength of the control matrix from 0.5575 to 0.2454. The study generated lesioned network from the connectivity matrix generated from the controls.

Complexity measures were estimated from the real EEG signals of patients and controls and simulated EEG signals of patients, controls, and lesion-induced control signals.

### Complexity Measures

Electroencephalogram complexity has been studied in the context of different neurological disorders and in healthy controls to gain insights into the dynamical property of the brain. LZC, HFD, and FDispEn had provided reliable conclusions in studies related to neurological diseases. LZC estimates the compressibility of EEG data and HFD measures fractal characteristics in EEG data. FDispEn estimates the uncertainty of the signal through the difference between the adjacent elements of the dispersion pattern.

#### Lempel-Ziv Complexity

Lempel-Ziv complexity is derived from the compressibility of the binary data (Lempel and Ziv, 1976). This measure could reveal the regularity and randomness in high-dimensional non-linear systems and is widely used in biomedical applications (Aboy et al., 2006). EEG signals are binarized using a threshold level and then analyzed for LZC. The median value in the EEG data sample is selected as the threshold level. Data sample above the threshold is equated to 1 and below the threshold level to 0. The resulting binary segment is scanned for different patterns. The counter *c*(*n*) is increased by one unit when a new pattern is encountered in the scanning process (Zhang et al., 2001).


(5) $$\begin{array}{r} {lim}_{n\to \infty }c\left(n\right)\;=\;b\left(n\right)\;=\;\frac{n}{\log_{2}n} \end{array}$$

In this equation, *n* is the length of the binary sequence and *b*(*n*) provides the upper bound of *c*(*n*). *c*(*n*) is normalized as follows:


(6) $$\begin{array}{r} \text{C}\left(\text{n}\right)\;=\;\frac{\text{c}\left(\text{n}\right)}{\text{b}\left(\text{n}\right)} \end{array}$$

After normalization, the complexity measure (*C*(*n*)) reflects the rate of occurrences of new patterns with an increase in time.

#### Higuchi's Fractal Dimension

Higuchi's fractal dimension measures the self-similarity (scale-free behavior) of a system. In a time-series data, FD could range from 1 to 2, with a higher value indicating higher signal complexity. EEG data show fractal properties with statistical similarity at different time scales. In this study, the fractal dimension algorithm has been selected as it provides a good approximation of fractal dimension in EEG signals. The algorithm uses a small number of data points to approximate the mean length of the curve. HFD had been successfully utilized by Gómez et al. (2009) and Smits et al. (2016) in order to analyze EEG.

An EEG signal [*y*(1), *y*(2), ……..*y*(*N*)] with a sample length of *N* can be divided into *k* length sub-data as


(7) $$\begin{array}{r} {\text{y}}_{\text{k}}^{\text{m}}:\text{y}\left(\text{m}\right),\text{y}\left(\text{m}+\text{k}\right),\text{y}\left(\text{m}+2\text{k}\right),\ldots \ldots ..\text{y}\left(\text{m}+\text{int}\left[\frac{\text{N}-\text{m}}{\text{k}}\right]\text{k}\right) \end{array}$$

In this equation, *k* is constant, and *m* = 1, 2, ….*k*. The [ ] is the Gauss' notation and length *L*_*m*_(*k*) of each curve $y_{k}^{m}$ is calculated as follows:


(8) $${\text{L}}_{\text{m}}\left(\text{k}\right)\text{​}=\text{​}\frac{1}{\text{k}}\text{​}\left[\text{​}\frac{\text{N}-1}{\text{int}\left[\frac{\text{N}-\text{m}}{\text{k}}\right]\text{k}}\text{​}\left(\text{​}\sum_{\text{i}\;=\;1}^{\text{int}\left[\frac{\text{N}-\text{m}}{\text{k}}\right]}\left|\text{y}\left(\text{m}+\text{ik}\right)-\text{y}\left(\text{m}+(\text{i}-1)\text{k}\right)\right|\text{​}\right)\text{​}\right]$$

The mean of *L*_*m*_(*k*) is computed to estimate HFD.


(9) $$\begin{array}{r} HFD\;=\;\frac{1}{k}\overset{k}{\underset{m\;=\;1}{\sum }}L_{m}\left(k\right) \end{array}$$

For the HFD calculation, *k* involved in the estimation was optimized at 18 and 6 for real and simulated EEG signals, respectively.

#### Fluctuation-Based Dispersion Entropy

Fluctuation-based dispersion entropy is a recent approach based on Shannon entropy and symbolic dynamics (Azami and Escudero, 2018). It is an efficient method to measure dynamic variability in real-time neurological data. The measure is relatively faster, insensitive to noise, and detects simultaneous amplitude and phase variations. Dispersion entropy (DispEn) uses a mapping function that transforms the EEG data to a new time series data of symbolic sequences with fewer elements (Azami and Escudero, 2018; Nieto-Del-amor et al., 2021). It estimates the regularity of the patterns with similar dispersion patterns. FDispEn captures the difference between adjacent elements of the dispersion pattern.

Algorithm for the FDispEn calculation for a given univariate data sample *y*_*j*_(*j* = 1……*N*) of length *N* is as follows:

1. The time series *y*_*j*_ is mapped with a mapping function to c classes. The classes are labeled as 1–c. A number of linear and non-linear mapping functions can be utilized for this process. Each sample is grouped to its nearest class based on its amplitude. A classified signal *u*_*j*_(*j* = 1……*N*) is thus obtained.
2. With an embedding dimension (*m*), and time delay (*d*) multiple time series, of length m, $u_{i}^{m,c}\;=\;\left\{u_{i}^{c},u_{i+d}^{c},\ldots \ldots u_{i+\left(m-1\right)d}^{c}\right\}$ for each *i* = 1, 2, …..*N*−(*m*−1)*d* are generated. Each $u_{i}^{m,c}$ is mapped to its dispersion pattern (Azami and Escudero, 2018). The number of possible dispersion patterns for each $u_{i}^{m,c}$ is *c*^*m*^.
3. FDispEn calculates the difference between adjacent dispersion patterns. For a vector length of *m*−1, each element changes from –*c*+1 to *c*−1. Thus, the number of fluctuation-based dispersion patterns for each $u_{i}^{m,c}$ is (2*c*−1)^*m*−1^.
4. The relative frequency of each (2*c*−1)^*m*−1^ dispersion patterns is calculated. It is used for the calculation of the FDispEn value of the input time series based on Shannon's entropy.

The study used *m* = 3 and *c* = 3 as the embedding dimension and number of classes, respectively, for the estimation of FDispEn (Azami and Escudero, 2018).

## Statistics

The Student's *t*-test has been used to investigate the significant difference between the patient and the control group. Normality in the data was ensured using the Shapiro-Wilk test. As the results from simulation experiments did not meet parametric assumptions, the Wilcoxon rank-sum (Mann-Whitney) test, a non-parametric test, has been used for simulated EEG signals. A false discovery rate (FDR) correction was applied across multiple comparison studies.

## Results

In this study, the complexity analysis was carried out in real and simulated EEG signals to explore the relation between functional connectivity and complexity in the context of MCI-AD under resting eyes open conditions. The mean connectivity strength of control, patient, and lesion-induced connectivity matrices was 0.5575, 0.4945, and 0.2454, respectively. The study performed three statistical investigations, namely, (1) comparison between real EEG signals of MCI-AD and healthy controls, 2) comparison between simulated EEG signals of patients with MCI-AD and healthy controls, and 3) comparison between simulated EEG signals from control and lesion-induced control. Results from these analyses are described in the following subsections. The ANOVA test conducted between real and simulated EEG signals displayed a significant difference of *p* = 0.0001.

### Comparison Between Real EEG Signals of Patients With MCI-AD and Healthy Controls

**Figure 3:**
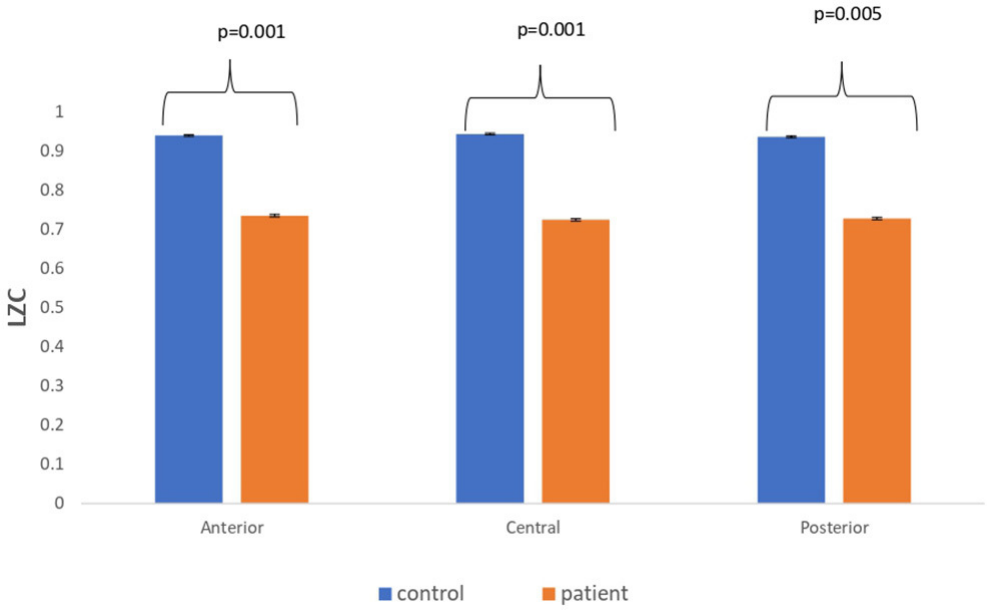
A bar chart plot of mean LZC values of real EEG signals of patients with MCI-AD and controls computed for anterior, central, and posterior regions.

**Figure 4:**
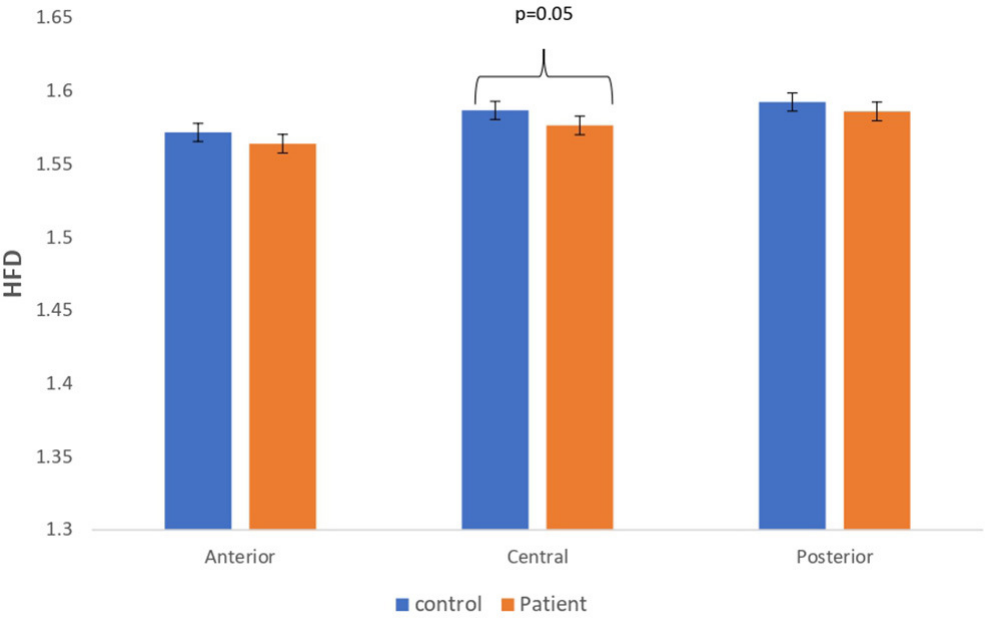
A bar chart plot of mean HFD values of real EEG signals of patients with MCI-AD and controls computed for anterior, central, and posterior regions.

**Figure 5:**
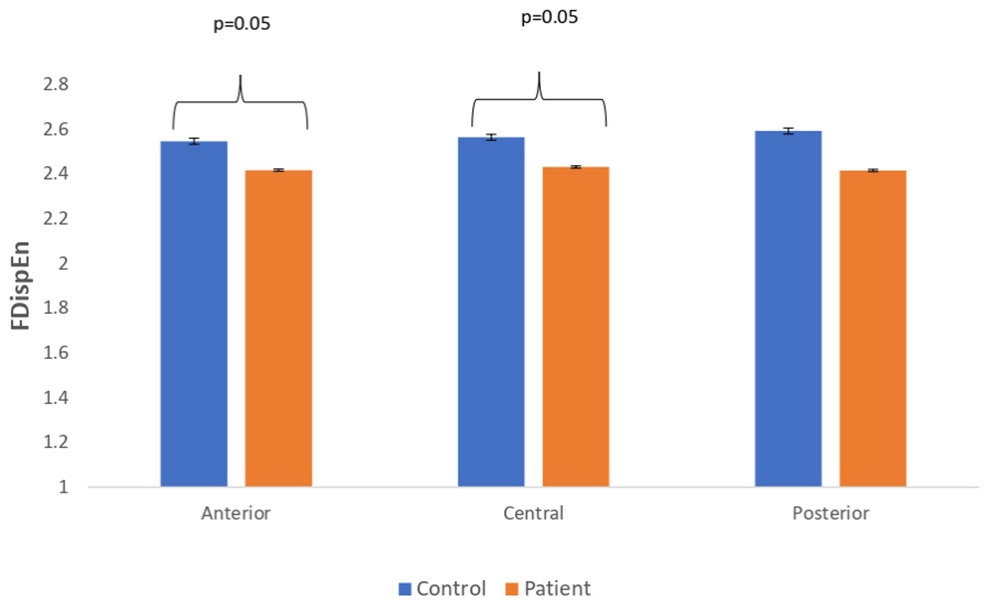
A bar chart plot of mean FDispEn values of real EEG signals of patients with MCI-AD and controls computed for anterior, central, and posterior regions.

The complexity measures of LZC, HFD, and FDispEn were used to analyze EEG signals of patients with MCI-AD and healthy controls. It was observed from the analysis of all the three complexity measures that the patients with MCI-AD have reduced complexity with respect to the control group. Reduction in complexity was displayed in anterior (*p* = 0.001), posterior (*p* = 0.005), and central (*p* = 0.001) regions for the LZC measure. However, significantly reduced complexity was observed only in the central region (*p* = 0.05), employing the HFD metric. Lower values of the FDispEn value were obtained for the anterior (*p* = 0.05) and central (*p* = 0.05) regions. Bar chart plots for the values computed for LZC, HFD, and FDispEn for patients with MCI-AD and controls across the three regions are displayed in Figures 3–5, respectively. It is observed from the plots that the MCI-AD condition is accompanied by a reduction in EEG complexity. Additionally, the central region of the MCI-AD brain has displayed reduced complexity for all the EEG complexity measures employed.

### Comparison Between Simulated EEG Signals of Patients With MCI-AD and Healthy Controls

**Figure 6:**
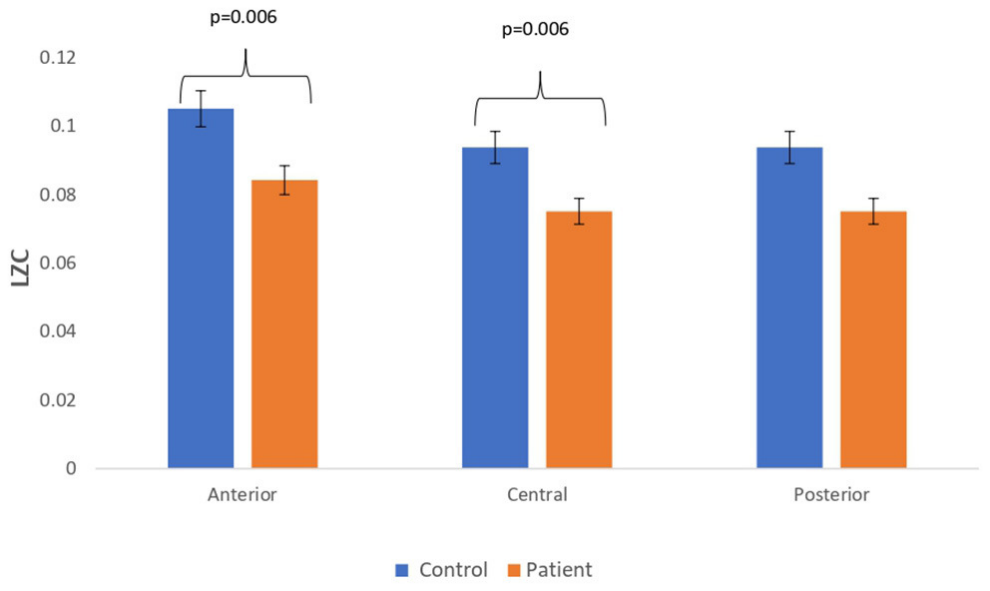
Mean LZC values calculated from simulated EEG data channels of patient and control group across anterior, central, and posterior regions.

Complexity measures are calculated from simulated EEG signals generated from the Kuramoto mean-field model utilizing connectivity network of patients and controls. The analysis has revealed a reduction in complexity estimates in simulated EEG signals of patients with respect to that of the simulated EEG signals from controls. A bar chart plot of mean values of LZC complexity measured across three regions, anterior, central, and posterior, is shown in Figure 6. A significant reduction in the complexity score was observed for the anterior (*p* = 0.006) and central (*p* = 0.006) regions. However, a significant difference was not visible for HFD and FDispEn measures. The reduction in complexity measure on simulated patient EEG channels indicated that the signals have become more regular and less complex than the simulated control EEG signals. This study indicated that lesser functional connectivity registered in patient matrix leads to a reduced patient EEG complexity estimate with respect to control. The results thus imply that connectivity between the limit cycle oscillator in the Kuramoto model has a direct effect on complexity values in the subsequently generated signal.

### Comparison Study Between Simulated EEG Signals of Controls and Lesion-Induced Control Signals

**Figure 7:**
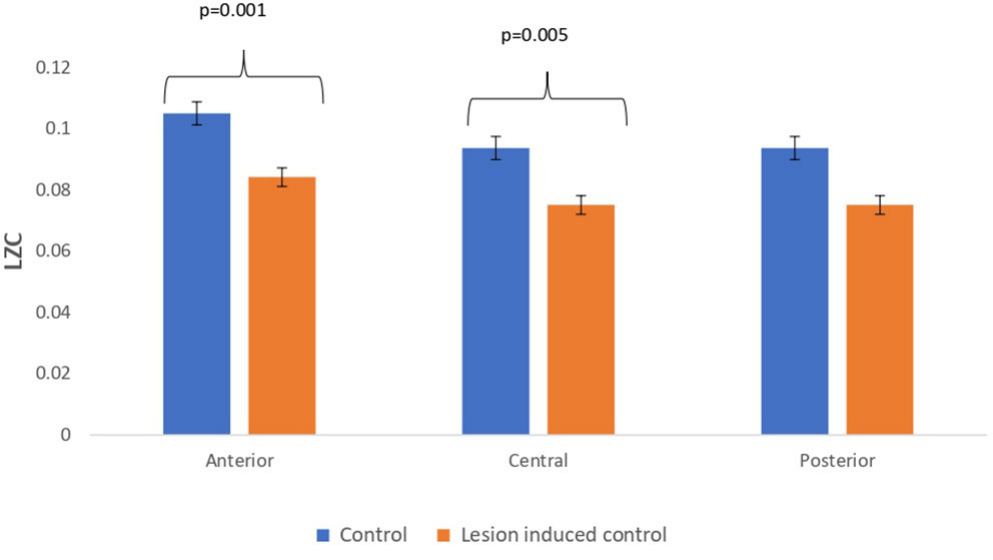
Mean LZC values calculated from simulated EEG data channels of control and lesion-induced control signals across anterior, central, and posterior regions.

**Figure 8:**
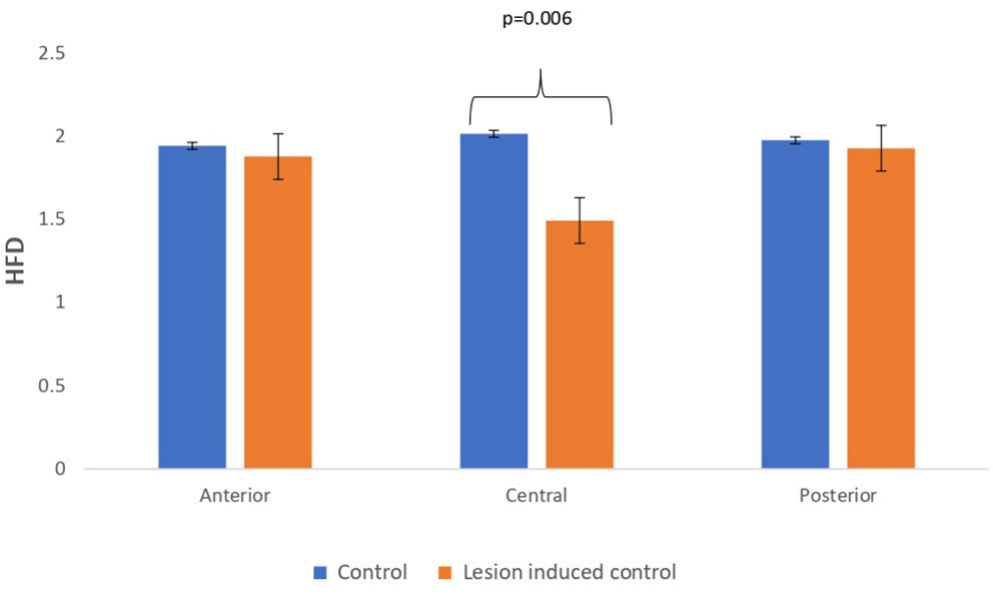
Mean HFD values calculated from simulated EEG data channels of control and lesion-induced control signals across anterior, central, and posterior regions.

**Figure 9:**
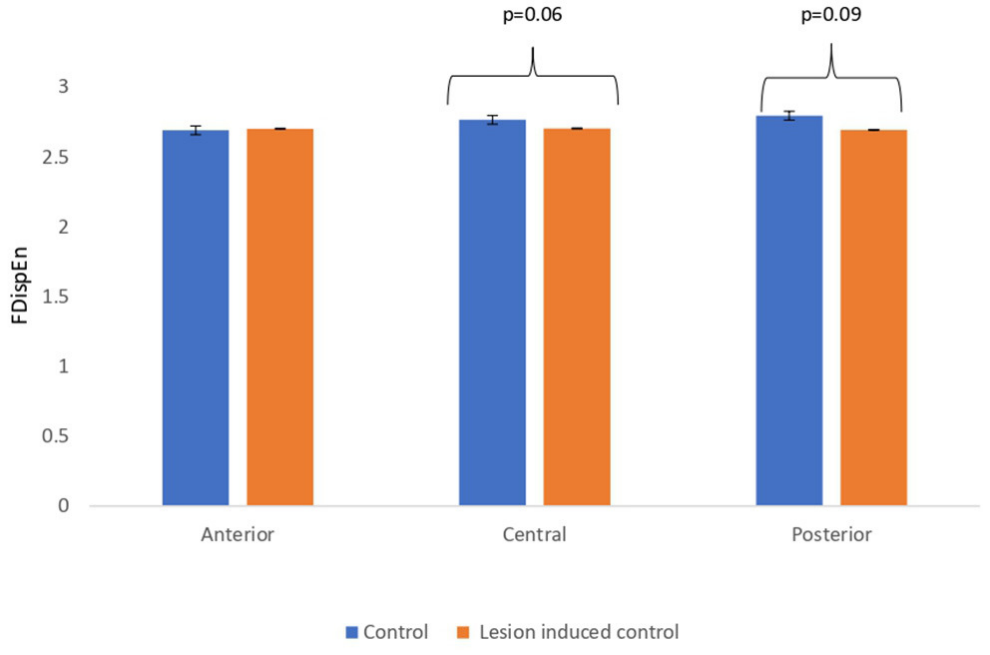
Mean FDispEn values calculated from simulated EEG data channels of control and lesion-induced control signals across anterior, central, and posterior regions.

Lesion-induced connectivity patterns were generated by replacing the connectivity score of the edges joining the central region by a value of 0.1. This would result in a transformed connectivity pattern with lowered connectivity score. A comparative study was conducted between simulated EEG signals generated from control and lesion-induced control signals. The results revealed a reduction in complexity values in the simulated EEG signals obtained from the lesion-induced control connectivity matrix in comparison with the simulated EEG signals generated from the connectivity matrix of the controls. Bar chart plots of mean complexity values of LZC, HFD, and FDispEn measures from control and lesion-induced control signals are shown in Figures 7–9, respectively. A significant difference was observed for LZC values in anterior (*p* = 0.001) and central (*p* = 0.005) regions. Reduced values of HFD were obtained for the central region (*p* = 0.006) with no significant statistical difference in the anterior and posterior regions. FDispEn has revealed differences in the central (*p* = 0.06) and posterior (*p* = 0.09) regions. From the analysis, it is apparent that the introduced lesion has induced a significant reduction in complexity values.

The EEG complexity analysis carried out on the signals from patients with MCI-AD and controls revealed a reduction in complexity in the patient group with respect to the controls. Results from the comparative study between simulated EEG signals of patients with MCI-AD and healthy controls and between simulated EEG signals from control and lesion-induced control, ascertain a positive relation between reduced EEG complexity to reduced connectivity pattern.

## Discussion

In this study, the relation between two prominent features, namely, functional connectivity and complexity of MCI-AD, are explored. The study investigates the influence of brain connectivity on EEG complexity by employing a phenomenological model of MCI-AD. The study employed functional connectivity matrix of patients with MCI-AD and the control group to generate simulated EEG signals using the Kuramoto mean-field model. Complexity measures are calculated from real and simulated EEG signals. The study also explored the effect of an induced lesion in the connectivity pattern and its resultant effect on complexity values. The functional connectivity matrix is calculated using WPLI. Popularly used complexity measures in EEG signal analysis such as LZC and HFD together with the novel entropy measure of fluctuation-based dispersion were used for EEG signal analysis.

Complexity analysis is performed to understand the amount of uncertainty or irregularity in data. The complexity aspect of neurological data has been explored with the hypothesis that complexity in the data indicates the adaptability of the system to function in varying environments. It further hypothesizes that the effect of a pathological process could hamper adaptability and would be reflected in the complexity estimates. The brain may exhibit increased or reduced complexity as a result of underlying pathology. Deviation in the mean complexity score observed in the patient group in comparison to a healthy control population could be the result of the detrimental effects of the underlying condition. Complexity studies conducted in the brain have shown this deviation with an increase in the complexity in the case of schizophrenia (Takahashi et al., 2010; Fernández et al., 2013; Ibáñez-molina et al., 2018), in normal aging (Anokhin et al., 1996) and a decrease in the complexity with MCI-AD in this study. In this study, the complexity scores of the patient were compared with that of the control group to study the deviation. The study reveals that alternations in the complexity estimates have their onset in the early phases of AD. All the three complexity measures of LZC, HFD, and FDispEn revealed a reduction in complexity in the EEG signals in patients with MCI-AD in comparison to the control group.

The reduction of EEG complexity estimates in patients is an indication of the EEG waveforms becoming more regular and less complex. It is suggestive of the possible effect of the MCI-AD-related pathological process, disintegrating the functional coupling and enabling neuronal bodies to behave more independently generating simple predictable waveforms (Jeong, 2004; Dauwels and Cichocki, 2011). The study has further revealed that the reduction in complexity estimates was significant in the anterior and central regions of the MCI-AD brain. The result is in agreement with previous studies conducted in AD (Dauwels and Cichocki, 2011; Smits et al., 2016; Al-nuaimi et al., 2018; Nesma et al., 2018; Nobukawa et al., 2019) and in limited studies conducted in early AD (Labate et al., 2013; Zhu et al., 2017). Histopathological studies in early AD and AD have shown the presence of atrophy in the medial temporal lobe and the association cortices (Chetelata and Baron, 2003; Teipel et al., 2006). The evidence of change revealed in this study in the anterior and central regions is inclusive of frontal and temporal lobes that account for the memory and non-memory impairment observed in the prodromal phase of AD (Chetelata and Baron, 2003; Teipel et al., 2006; Dauwels and Cichocki, 2011).

Along with the reduction of complexity, reduction in functional connectivity is a hallmark feature of the EEG analysis in AD (Dauwels and Cichocki, 2011). The functional connectivity analysis in this study is carried out using the WPLI. The connectivity analysis based on WPLI revealed a reduction in mean connectivity strength in the patient group. Several electrophysiological studies have extensively explored AD-related changes revealing a reduction in connectivity (Babiloni et al., 2004; Engels et al., 2015; Afshari and Jalili, 2017; Triggiani et al., 2017) and complexity (Dauwels and Cichocki, 2011; Labate et al., 2013; Smits et al., 2016; Al-nuaimi et al., 2018; Nesma et al., 2018) discretely, without analyzing the influence of one over the other. The results from the current study disclose a positive influence between the two features of functional connectivity and complexity accomplished through the study of the Kuramoto model. A reduction in values of complexity estimates was observed for the simulated EEG signal from the patients with MCI-AD when matched with the controls. In addition, results from the simulated EEG analysis from lesion-induced controls and controls provide a similar inference. From the simulated EEG signals analysis, it was observed that reduced complexity has been consecutively associated with the group having a reduced connectivity score. Simulated EEG signal from lesion-induced control is an attempt to simulate the effect of the discontinuous network in MCI-AD. The Kuramoto model could generate a more disconnected set of EEG signals by reducing the connectivity strength from the edges originating from the central region of the connectivity matrix. Thus, from the comparative studies, it is evident that the functional connectivity matrix holds intricate relation with the signal complexity estimated.

The relation between connectivity and complexity could be understood with the help of the meta-stability concept. The meta-stability concept in the brain provides the theoretical foundation to explain how complex features emerge and are capable of information processing, data transmission, and storage. The Kuramoto model essentially describes a phase model that can exhibit spontaneous translations from random incoherent phases to collective synchrony as the coupling parameter passes through a critical threshold value. The coupling parameter in the metastable region allows the model to simulate data that resemble brain data with features of spontaneous transition between multiple transient states. The reduction in the connectivity/coupling in the coupled system of oscillators could have enabled the individual oscillators to behave more incoherently, thus reducing the “meaningful structural richness” (Costa et al., 2005) of the simulated signal. Accordingly, the reduction in the complexity could be related to the decline in the capability of the system to visit a wide repertoire of possible states, thus affecting the adaptability of the brain to varying environmental conditions.

Several complexity measures have been applied to study EEG signals over the years. The distinction between meaningful structural richness and randomness in the system remains unclear as both systems are capable of generating unpredictable and irregular signals. Complexity is defined as an intermediate stage between randomness and order. Complexity measures used in this study, LZC, HFD, and FdispEn, capture different aspects of the system dynamics. LZC and FDispEn measure the regularity index of a dynamical system through the amount of the uncertainty element. FDispEn is based on Shannon entropy and estimates dynamical variability through dispersion patterns. LZC estimates are based on scanning the symbolic representation of time series data for new patterns. It is a useful means to estimate the bandwidth of random process and harmonic variability in a quasi-periodic signal (Aboy et al., 2006). HFD captures the signal at different scales and investigates the self-similarity in the time series data. HFD is insensitive to stereotypical or repetitive signals. Therefore, it is possible to have a signal with a low LZC value with high HFD if the signal is a disordered signal composed of similar patterns (Jos et al., 2018). The complexity measures LZC, HFD, and FDispEn are capable of measuring certain aspects of “structural richness” in the signal. From the results of simulation studies, it is discerned that the regional complexity score is influenced by the introduction of lesion in the connectivity matrix. Thus, the reduction in coordination with multiple coupling strength in the simulation of EEG signal could have resulted in reduction in “meaningful structural richness” or complexity.

Mixed patterns of both positive (Nobukawa et al., 2020) and negative correlation (Mcdonough and Nashiro, 2014; Jos et al., 2018) between connectivity and complexity have been reported in few studies conducted on neurophysiological and simulated data. A recent study observed a positive correlation between connectivity and complexity in AD (Nobukawa et al., 2020). The current study supports this result with an abstract modeling approach to the MCI-AD condition. However, it should be noted that it is yet to be fully understood whether the reduction of connectivity and complexity could be the result of a direct association or is the by-product of diverse neurological activities in the MCI-AD brain. One of the major limitations of the study is that the Kuramoto model used in the study is a fairly simple model and would not be able to reconstruct all the characteristic features of the EEG signal. Furthermore, this model used a limited number of oscillators to simulate the signal. Despite this, the comparison analysis by using the complexity analysis showed that the model could fairly simulate EEG signals at a similar level of complexity as a real EEG signal. This study suggests that the reduction of functional integration between brain regions caused by the loss of connectivity could be one of the possible reasons for the reduction of richness or complexity in the EEG signals. Future studies could use the results from this study to understand the neurodynamics behind the electrophysiological observation in EEG under MCI-AD conditions.

## Conclusion

The study attempted to analyze the relation between functional connectivity and complexity by modeling the MCI-AD condition with the help of the Kuramoto model. EEG signals from the MCI-AD condition have shown altered neurodynamics, displaying a reduction in the estimates of connectivity and complexity. From the studies using the Kuramato model, it was found that the connectivity of the coupled oscillators has a direct influence on the complexity of the generated signal. A significant observation from the results of the study is the possible direct influence of reduced connectivity between brain regions in lowering the complexity score of the EEG signal.

## Data Availability Statement

The datasets presented in this article are not readily available because the data is protected by a copyright that restricts sharing. Requests to access the datasets should be directed to SP, subhadp@nitc.ac.in.

## Ethics Statement

The studies involving human participants were reviewed and approved by the research and ethical committee of Sree Chithra Tirunal Institute of Medical Sciences and Technology, Trivandrum, Kerala, India. The patients/participants provided their written informed consent to participate in this study.

## Author Contributions

All authors listed have made a substantial, direct, and intellectual contribution to the work and approved itfor publication.

## Conflict of Interest

The authors declare that the research was conducted in the absence of any commercial or financial relationships that could be construed as a potential conflict of interest.

## Publisher's Note

All claims expressed in this article are solely those of the authors and do not necessarily represent those of their affiliated organizations, or those of the publisher, the editors and the reviewers. Any product that may be evaluated in this article, or claim that may be made by its manufacturer, is not guaranteed or endorsed by the publisher.
